# Naturalistic Online Language as a Marker of Depression in Midlife and Older Adults: Computational Text Analysis of Bluesky and Reddit Posts

**DOI:** 10.2196/95023

**Published:** 2026-08-26

**Authors:** Lauren Rutter, Andy Edinger, Marijn ten Thij, Lorenzo Lorenzo-Luaces, Danny Valdez, Johan Bollen

**Affiliations:** 1 Department of Psychological and Brain Sciences, Indiana University, 1101 E 10th Street, Bloomington, IN, 47405, United States, 1 (812) 856-9953; 2 Indiana Alzheimer’s Disease Research Center, Indiana University School of Medicine, Indianapolis, IN, United States; 3 Cognitive Science Program, Indiana University, Bloomington, IN; 4 Department of Intelligent Systems, Tilburg University, Tilburg, North Brabant, The Netherlands; 5 Department of Applied Health Sciences, Indiana University, Bloomington, IN; 6 Informatics Institute, Faculty of Science, University of Amsterdam, Amsterdam, North Holland, The Netherlands; 7 Department of Informatics, Computing, and Engineering , Luddy School of Informatics, Computing, and Engineering, Indiana University, IN, United States

**Keywords:** naturalistic language, older adults, depression, midlife adults, cognitive distortions

## Abstract

**Background:**

Social media use among older adults continues to grow. Many people use social media to establish meaningful social ties and discuss their mental health. Depression in midlife and older adults is a critical public health concern, yet scalable, sensitive methods for early detection remain limited. Natural language processing offers new opportunities to examine sentiment and mental health through online language in typically understudied populations.

**Objective:**

This study examined whether sentiment and cognitive features of naturalistic social media language are associated with depression diagnosis and symptom severity among midlife and older adults.

**Methods:**

We constructed cohorts of adults aged 50 years and older from Reddit (n=688) and Bluesky (n=210). Participants who had ever had depression and never been diagnosed were recruited via a self-report survey on Prolific that collected depression history, age of diagnosis, current symptoms, and social media handles. Public posts were preprocessed and analyzed using a validated rule-based sentiment analysis (Valence Aware Dictionary and Sentiment Reasoner). Sentiment measures were aggregated at the user level and compared by depression status within and across platforms.

**Results:**

On Bluesky, users with a history of depression exhibited lower Valence Aware Dictionary and Sentiment Reasoner compound sentiment compared to users who had never been diagnosed (ρ=−0.16; *P*<.001). On Reddit, current depression severity showed a small association with lower compound sentiment, and although the bootstrap CI excluded zero, the association did not remain statistically significant after correction for multiple comparisons (*P*>.05). These findings indicate a platform-specific relationship between depression status and online sentiment.

**Conclusions:**

Negative sentiment expressed on Bluesky was associated with depression history (ever diagnosed vs never diagnosed) among midlife and older adults. On Reddit, current depression severity showed a small association with negative sentiment that did not remain statistically significant after correction for multiple comparisons. Our findings suggest that sentiment-based language features may capture modest differences in online expression related to depression. Future work with larger samples and longitudinal symptom assessment is needed to determine whether sentiment reliably tracks with depression-related symptoms over time.

## Introduction

Over the past decade, digital traces from online platforms have become a dominant source of data, enabling researchers to study mental health phenomena naturalistically and at scale. Social media has emerged as a promising tool to understand processes related to emotions and mental health, offering insights into sleep [1], social relationships [2], markers of distress, and trajectories of well-being over time [3-6]. While activity on social media platforms has historically been dominated by young adults, there has been a steady increase in online engagement among midlife and older adults [7]. On the basis of a 2025 Pew Research Center survey, 90% of adults aged 50 to 64 years and 78% of adults aged 65 years and older own a smartphone [8]. As digital participation rises among midlife and older adults, so too does the opportunity to harness these platforms to assess psychological risk and identify early warning signs of disorders such as depression.

Prior work suggests that smartphone and social media use among older adults is relevant for social connection, loneliness, well-being, and mental health [9-14]. In a study of social media use among adults aged 65 years and older, active and passive social media use was differentially associated with depressive symptoms, with active use increasing odds of depressive symptoms and passive use lowering odds [10]. In another study of smartphone use in older adults, smartphones were used for social and nonsocial purposes, with social media and reading the news as the most common activities [11]. Social media is increasingly used not only to connect with others but also to disclose health experiences, including symptoms of depression, diagnoses, stress, and related concerns [15-17]. This is important because an estimated 10% to 15% of older adults have depressive symptoms. Depression symptoms, even those that are mild, are distressing [18,19]. At the same time, adults in midlife remain less represented in social media–based mental health research. Studying adults aged 50 years and older provides an opportunity to examine whether computational markers of depression extend to midlife and older adult populations—populations that are both increasingly online and relevant to the study of depression in later life.

Natural language processing (NLP), deep learning, and other computational methods offer powerful tools for extracting information about mental health on social media at scale. In prior work, we extracted the hourly number of Twitter (subsequently rebranded X) posts from a large sample of users as an index of overall activity (ie, behavior) [1]. Content analyses revealed that the sample with depression showed greater levels of positive and negative emotionality when active and a steady rise in ruminative language and rigid thinking from midnight to dawn. In a follow-up study [20], we developed a theory-driven cognitive distortion schemata (CDS) lexicon to capture language reflecting negatively biased or rigid thinking patterns that are central in cognitive models of depression. We refer to the CDS lexicon as theory-driven because it is based in cognitive theories of depression, particularly those articulated by Beck and Haigh [21]. In contrast to data-driven lexicons that are created from words that statistically distinguish groups or symptom-based depression lexicons that focus on depressive content, the CDS lexicon was designed to capture linguistic patterns consistent with theoretically defined cognitive distortions. Cognitive distortions refer to maladaptive thinking patterns that are negatively biased interpretations of the self, world, and future. These patterns include all-or-nothing thinking (eg, “Anything less than 100% is failure”), jumping to conclusions (eg, “The email from my boss requesting a meeting means I did something wrong”), catastrophizing (eg, “Because he didn’t text me back, he probably doesn’t care about me—I will be single and die alone”), and emotional reasoning (eg, “Because I feel anxious, I won’t have fun tonight”), for example. Using this lexicon, we found in our previous Twitter study that cognitive distortion language was significantly more prevalent in the messages posted by users with depression compared to a random sample, which was true across all age categories. Additional work using this CDS lexicon has examined broader temporal trends in cognitively distorted language and shown that there has been a rise in written distorted language in recent decades [22,23].

Previous research has shown that detailed indicators of behavior [1,24], mood and affect [25], cognitive vulnerability [26,27], and social relationships [28] can be extracted from social media content for millions of individuals. While our prior work used Twitter in all ages, in this study, we extended our work to target adults aged 50 years and older who were active on Reddit, a long-form social networking website, and Bluesky, a short-form social networking website with similar functionality to that of X. We created a novel dataset of Reddit and Bluesky users aged 50 years and older who reported a lifetime diagnosis of depression and an age-matched never-diagnosed comparison group. Our prior work validated the CDS lexicon using Twitter; however, changes in X API access limited our ability to collect new data from the populations of interest. Therefore, we selected Bluesky as a short-form alternative with similar functionality to that of X. Reddit was selected as a contrasting text-based platform. Publicly available posts were retrieved using the Bluesky API and Reddit API through user handles. Data collection was conducted in accordance with the platforms’ applicable terms and institutional review board approval. Together, Reddit and Bluesky allowed us to examine naturalistic language across two distinct, text-based social media platforms.

This study addressed 2 research questions. First, we examined whether adults aged 50 years and older with a lifetime diagnosis of depression differed from an age-matched never-diagnosed comparison group in sentiment across Reddit and Bluesky. As a second, exploratory question, we examined whether depression diagnosis was related to cognitive distortion prevalence in adults aged 50 years and older. Prior work has linked depression with more negative sentiment and greater use of cognitive distortion language [29], but it remains unclear whether these patterns extend to midlife and older adults on Reddit and Bluesky. By examining sentiment and cognitive distortion language in naturalistic posts across two public, text-based social media platforms, this study addressed gaps in the literature on depression-related language in a digitally active but understudied age group.

## Methods

### Ethical Considerations

This research was reviewed by the Indiana University Bloomington Institutional Review Board. The broader grant under which this work was conducted was approved under protocol 21317, and the Prolific study reported here was reviewed under protocol 27266 and determined to be exempt research. Participants recruited through Prolific were provided with a study information sheet describing the research prior to participation, and a signed informed consent form was not required.

### Data Privacy and Handling

We aimed to recruit social media users with depression who were aged 50 years and above and a comparison group of users aged 50 years and older who had never had depression. As described below, we recruited users with depression from a Prolific study called “Seeking Social Media Users with Diagnosed Depression.” Prolific is an online research participant platform. Prolific users were recruited between May 2025 and September 2025.

To protect participant privacy, social media usernames and handles were removed from the analytic post files and were not stored with age, sex, or depression status information. Posts were saved as text files grouped by depression history without usernames or demographic information included. Because social media posts may contain self-disclosed personal information, we refer to these files as deidentified rather than anonymized. This study was not preregistered.

### Cohort Construction

We identified users who had ever had depression and never had depression through a Prolific study asking individuals aged 50 years and older who used social media to fill out a short self-report survey about whether they had ever been diagnosed with depression and their current depression symptoms. Subsequently, we asked them to provide their Reddit and Bluesky handles. From the provided social media handles, we retrieved the available posting history for each user profile. We removed cases where individuals had no posts or provided invalid usernames. Non–English-language posts were excluded, and reposts were removed as these were not concordant with our prior approach and did not reflect the individual poster’s original thoughts. For each account, we retrieved all posts available throughout the time of data collection. Our final analytic sample consisted of 154 users who had ever had depression diagnoses and 56 users who had never had depression diagnoses from Bluesky and 506 users who had ever had depression diagnoses and 182 users who had never had depression diagnoses from Reddit.

### Sentiment Analysis

Sentiment analysis refers to approaches that measure the valence or emotional tone of text using NLP. In this work, we used the Valence Aware Dictionary and Sentiment Reasoner (VADER) [30] to estimate the sentiment of posts. VADER is a rule-based sentiment tool developed specifically for social media text, incorporating a large lexicon of common words, abbreviations, and idiomatic expressions along with heuristics that account for punctuation, negation, hedging, and intensification. We have previously used VADER to study sentiment in social media posts of adults with depression and anxiety [4,20,29]. VADER assigns each word with emotional content a sentiment valence using a validated lexicon and then adjusts these values based on linguistic context, including negation, degree modifiers such as intensifiers or diminishers, punctuation, capitalization, and emojis. VADER computes a compound score, which ranges from –1, indicating maximally negative sentiment, to +1, indicating maximally positive sentiment. In the present analyses, the compound score was used as the primary indicator of overall sentiment, with lower values reflecting more negative sentiment.

### Cognitive Distortions

The conceptualization and assessment of cognitive distortion language is described in detail in prior work [20]. Because the current study applied the previously developed CDS lexicon, portions of our description are adapted from the prior publication. The Committee on Publication Ethics and Text Recycling Research Project describes text recycling as the reuse of material from an author’s prior work in a new document, including prose, visuals, or equations. In the current manuscript, any reused text is limited to methodological description of the previously developed CDS lexicon.

The CDS lexicon was developed to identify short words and word sequences that mark the building blocks of cognitive distortions as described in cognitive and cognitive behavioral models of depression. The categories of cognitive distortions that we chose included catastrophizing, dichotomous reasoning, disqualifying the positive, emotional reasoning, fortune-telling, labeling and mislabeling, magnification and minimization, mental filtering, overgeneralizing, personalizing, and “should” statements. In the original CDS development study [20], the lexicon was developed by a panel of psychologists with expertise in cognitive behavioral therapy, depression, cognitive distortions, and language-based assessment of depression. The panel engaged in a process of collaborative design followed by a consensus voting procedure requiring unanimous decision to identify 241 CDS n-grams ranging from 1-gram to 5-grams, each geared to express at least one type of cognitive distortion.

The candidate n-grams were selected to capture context-independent language patterns that could express distorted thinking, avoiding expressions that are specific to depression-related topics such as poor sleep, fatigue, or health issues. For example, the common 3-gram “I am a” was included as a building block of expressing a variety of labeling and mislabeling cognitive distortions because it would be a highly likely (and nearly unavoidable) n-gram to express many self-referential (“I”) expressions of labeling (“am a”). Where possible, higher-order n-grams were chosen to capture as much of the semantic structure of one or more distorted schemata as possible; for example, the 3-gram “everyone will believe” captures both overgeneralizing and fortune-telling. We did include 1-grams such as “nobody” and “everybody” as they strongly correspond to the expression of dichotomous reasoning. For the purposes of the current study, we combined all CDS categories to create an overall cognitive distortion language prevalence measure. Cognitive distortion prevalence was calculated by counting the number of posts that contained at least one CDS n-gram divided by the total number of posts, as described in the Data Analysis section.

### Depression Assessment

We used the Patient Health Questionnaire–8 (PHQ-8) [31], a widely used measure of depression symptoms with excellent psychometric properties. The PHQ-8 is an 8-item scale where 8 symptoms of depression are rated from 0 (“not at all”) to 3 (“nearly every day”). Scores range from 0 to 24, with 0 to 4 indicating no significant depression symptoms, 5 to 9 indicating mild depression, 10 to 14 indicating moderate depression, 15 to 19 indicating moderately severe depression, and 20 to 24 indicating severe depression. We selected the PHQ-8 because the study was conducted remotely without real-time clinical monitoring, and therefore, we did not administer the Patient Health Questionnaire–9 suicidal ideation item. Of note, PHQ-8 scores reflected depressive symptom severity during the 2 weeks before study enrollment and, therefore, may not reflect participants’ depressive symptom severity at the time their posts were written. Accordingly, associations between the PHQ-8 and language should be interpreted as exploratory examinations of the relationship between current depressive symptom severity and aggregated language history rather than evidence of contemporaneous associations between depressive symptoms and language use.

### Data Analysis

#### Overview

After creating our final analytic sample, described above in the Cohort Construction section, we normalized text across timelines by applying deterministic cleaning. Posts were lowercased; links, mentions, hashtags, and emojis were removed; white space was collapsed; and punctuation was normalized. We then conducted a user-level analysis for each cohort and compared emotional language and prevalence ratios of cognitive distortion between cohorts to test our hypotheses. We began by testing associations between variables using the Spearman correlation. Bluesky and Reddit users were kept as separate analytic groups. Cognitive distortion language was noted as present (1) or absent (0), and user-level analyses were conducted based on the following formula: user-level cognitive distortion prevalence = number of posts containing at least one CDS n-gram/total number of posts.

To quantify uncertainty in bivariate Spearman correlations and account for variance in posting volume across users, we applied bootstrap resampling (*k*=10,000 iterations) to all pairwise correlations as well as the linear model correlations. At each iteration, users were resampled with replacement, and Spearman correlations were computed across all variable pairs simultaneously. Bootstrap means and 95% CIs were derived from the resulting distributions (Tables S1 and S2 in Multimedia Appendix 1).

To further account for high variation in participant posting volume, we reran Spearman correlations between depression indicators (PHQ-8 total score and diagnosis status) and linguistic markers (CDS prevalence and VADER compound sentiment) across a range of minimum post thresholds (1, 5, 10, 25, and 50 posts per user). Correlations were computed separately for the Bluesky and Reddit samples (Tables S5 and S6 in Multimedia Appendix 1). Associations between CDS prevalence and VADER components should be interpreted cautiously because both measures were derived from the same text and may partly reflect lexical or mathematical dependence rather than distinct psychological processes.

To address potential confounding by demographic factors, we additionally computed partial Spearman correlations between depression indicators (PHQ-8 score and diagnosis status) and linguistic markers (CDS prevalence and VADER compound sentiment) controlling for age and biological sex. Analyses were conducted separately for Bluesky and Reddit samples. *P* values were corrected for multiple comparisons using the false discovery rate (FDR) correction procedure across all partial correlation tests (*k*=8; Table S7 in Multimedia Appendix 1).

#### Linear Modeling of Bluesky User VADER Scores

After computing VADER sentiment scores for each post in the Bluesky dataset, we used linear models to fit each user’s sentiment scores as a function of time, defined as the time elapsed since the user’s first observed post. Models were fit independently for each user using ordinary least squares regression. Only users with at least 2 posts with valid time stamps were included in the analysis. From each model, we extracted the estimated sentiment slope, intercept, and coefficient of determination (*R*^2^) as summary measures of longitudinal sentiment. The slope parameter thus measures within-user trends in affect over time, whereas the intercept gives some indication of baseline affect.

## Results

**Figure 1.**
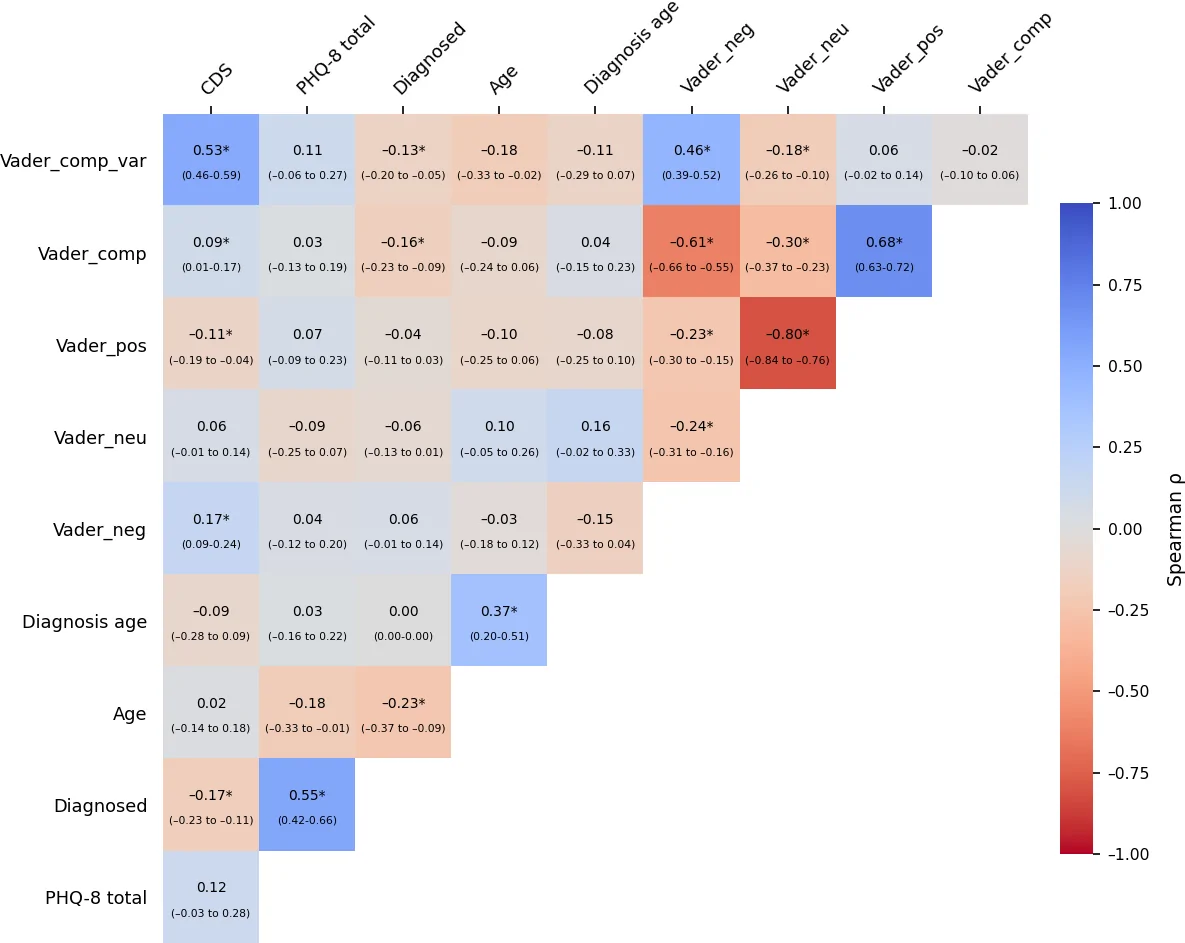
Pairwise Spearman correlation between natural language processing indexes and participant features in a sample of Bluesky users (n=210). CDS: cognitive distortion schemata; PHQ-8: Patient Health Questionnaire–8. *Correlations remained significant after false discovery rate correction.

**Figure 2.**
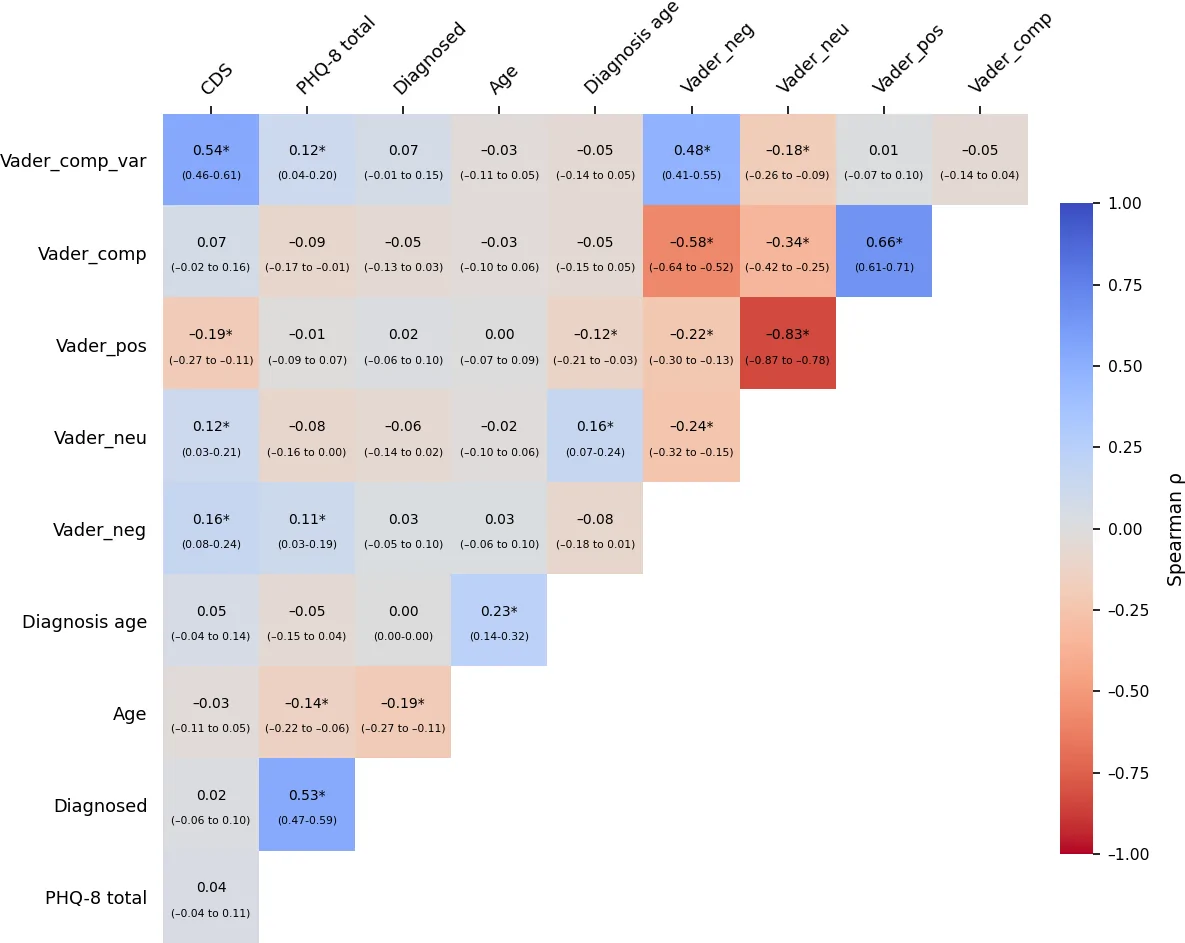
Pairwise Spearman correlation between natural language processing indexes and participant features in a sample of Reddit users (n=688). CDS: cognitive distortion schemata; PHQ-8: Patient Health Questionnaire–8. *Correlations remained significant after false discovery rate correction.

From Bluesky, our final analytic sample comprised 210 users with 117,801 posts. A total of 73.3% (154/210) of these users, with 97,031 posts, reported a depression diagnosis, whereas 26.7% (56/210) of the users, with 20,770 posts, reported no diagnosis. From Reddit, our final analytic sample comprised 688 users with 261,057 posts. Of these 688 users, 506 (73.5%) with 173,868 posts reported a prior diagnosis of depression, whereas 182 (26.5%) with 87,189 posts reported no depression diagnosis. Demographic characteristics of the analytic sample and descriptive statistics are shown in Table 1. Race and ethnicity data were not collected in the current study. To test our primary hypothesis that there would be differences in sentiment between groups who had ever had depression and never been diagnosed with depression, we examined the relationships between VADER and depression status and severity using Spearman correlations. Figure 1 and Figure 2 show the Bluesky and Reddit results, respectively, of heat mapped Spearman correlations with 10,000 bootstrap resampling and FDR comparisons applied.

**Table 1.** Descriptive information of the analytic sample of midlife and older adult Bluesky and Reddit users with and without depression (N=898).

	Bluesky	Reddit
Users, n (%)	210 (23.4)	688 (76.6)
Diagnosed, n (%)	154 (17.1)	506 (56.3)
Posts, mean (SD; range)	560.96 (3263.25; 1-44,697)	379.44 (669.63; 1-3850)
Age (y), mean (SD; range)	57.43 (5.82; 50-80)	56.72 (5.95; 50-80)
Diagnosis age (y), mean (SD; range)	36.10 (12.66; 12-66)	34.33 (12.73; 6-70)
PHQ-8^a^ total score (0-24), mean (SD; range)	8.26 (5.77; 0-24)	8.44 (6.12; 0-24)

a PHQ-8: Patient Health Questionnaire–8.

Results showed that, for the Bluesky dataset, there was a significant negative correlation between diagnosis status and VADER compound sentiment score: users who reported a previous diagnosis of depression were found to use more negative sentiment (ρ=−0.16; *P*<.001; Figure 1). However, while diagnostic status was associated with more negative overall sentiment, the proportional negative sentiment was not significant. This pattern was expected because the compound score captures the intensity and contextual weighting of negative language, whereas the negative score reflects only the relative frequency of negative tokens and can be diluted by neutral language. In the Reddit dataset, higher PHQ-8 scores were modestly associated with VADER compound sentiment in the unadjusted analysis (ρ=−0.09, bootstrap 95% CI −0.17 to −0.01; *P*=.03; Figure 2 and Table S4 in Multimedia Appendix 1); however, this association did not remain significant after FDR correction (*P*=.07) and is therefore interpreted as suggestive rather than statistically significant.

We next examined the correlations between sentiment and distorted thinking in our sample by conducting Spearman correlations to test the relationships between VADER scores and cognitive distortion use. The results were significant for positive and negative VADER scores on both Reddit and Bluesky and are shown in Table 2. Unexpectedly, on Bluesky, depression status was associated with lower CDS prevalence (ρ=−0.17; *P*<.001), which remained consistent across minimum post thresholds but was not significant after controlling for age and sex (Table S1 in Multimedia Appendix 1). For Reddit, CDS prevalence was not associated with diagnostic status (ρ=0.02; *P*=.64). CDS prevalence was not associated with PHQ-8 scores on either platform.

**Table 2.** Associations between sentiment (Valence Aware Dictionary and Sentiment Reasoner) and cognitive distortions in a sample of midlife and older adult social media users by platform sampled (N=898).

	Composite		Positive sentiment		Neutral sentiment		Negative sentiment	
	Spearman ρ	*P* value	Spearman ρ	*P* value	Spearman ρ	*P* value	Spearman ρ	*P* value
Reddit	−0.01	.82	−0.18	<.001	0.09	.03	0.17	<.001
Bluesky	0.00	.94	−0.12	<.001	0.04	.22	0.17	<.001

**Figure 3.**
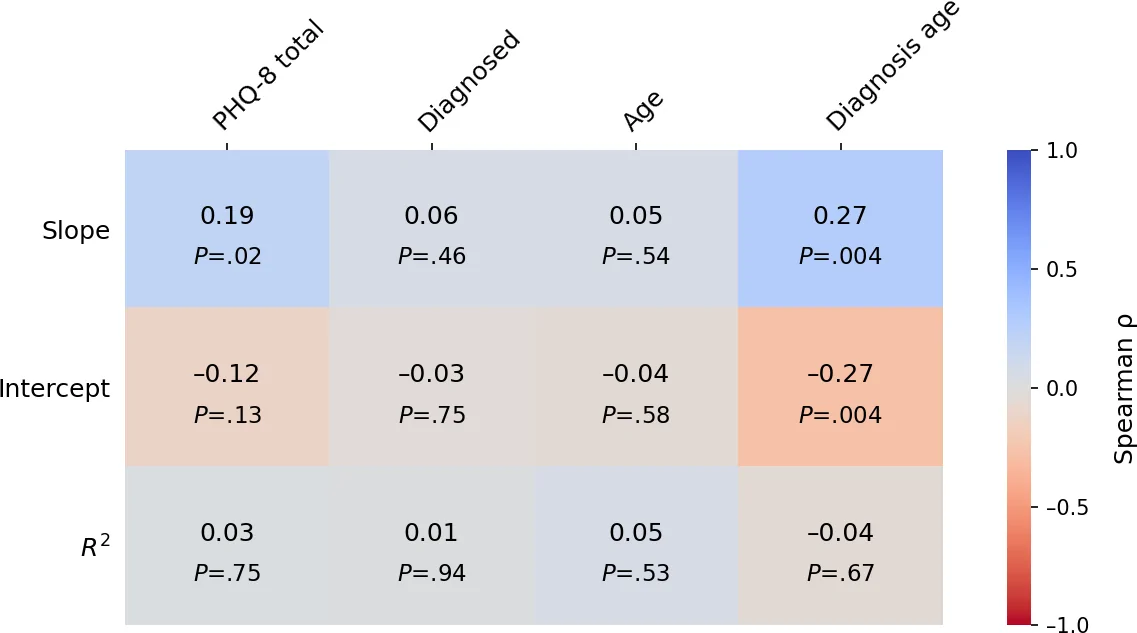
Spearman correlations between per-user linear model estimates of Valence Aware Dictionary and Sentiment Reasoner sentiment over time and participant depression-related features. PHQ-8: Patient Health Questionnaire–8.

To characterize longitudinal sentiment on Bluesky, we fit user-specific linear models of VADER sentiment scores as a function of time and examined associations between extracted model parameters and clinical and demographic variables (Figure 3). Comparable time-stamped data were not available for Reddit text files in the current study, and thus, longitudinal models could not be estimated for Reddit. For Bluesky, sentiment slopes were significantly associated with depressive symptom severity as measured using the PHQ-8 (ρ=0.19, bootstrap 95% CI 0.03-0.34; *P*=.02), indicating that individuals with higher depression symptom burden exhibited modestly more negative sentiment trajectories over time; however, this association did not remain statistically significant after FDR correction (*P*=.07) and should therefore be interpreted as suggestive and requiring replication in a larger sample. Sentiment slopes were also positively associated with age at depression diagnosis (ρ=0.27; *P*=.004). In contrast, baseline affect (intercept) was negatively associated with age at diagnosis (ρ=−0.27; *P*=.004), suggesting lower average sentiment among individuals diagnosed later in life. Diagnosis status itself was not significantly associated with sentiment slope (*P*=.46), intercept (*P*=.75), or model fit (*P*=.94). Coefficients of determination (*R*^2^) were small (Table S3 in Multimedia Appendix 1). Together, these findings suggest that longitudinal sentiment dynamics captured from social media are weakly related to current symptom severity and timing of diagnosis but not to diagnostic status per se, underscoring the importance of modeling sentiment patterns rather than relying on static clinical labels.

## Discussion

The online language of midlife and older adults on social media can offer insights into their health and social relationships, which are both important aspects of healthy aging [22,23]. In the current study, using the online language of midlife and older adults on Reddit and Bluesky, we compared a sample who had ever been diagnosed with depression to a never-diagnosed comparison sample in terms of VADER sentiment scores and prevalence of cognitive distortions across timelines. Results showed that, for Bluesky users in our sample, ever having had a depression diagnosis was associated with more negative sentiment compared to users who had never had depression. In Reddit data, depression severity but not diagnosis showed a small association with more negative sentiment in the unadjusted analysis, but this did not remain statistically significant after FDR correction. To the best of our knowledge, this is the first study to use Bluesky and Reddit to examine the emotional content of online language of adults aged 50 years and older using NLP in the context of depression. The results have implications for detecting linguistic features of depression and uncovering links among depression, language, and cognition in midlife and older adults.

Building on these findings, and given the associations among diagnosis age, symptom severity, and sentiment, we conducted additional exploratory analyses to examine whether longitudinal patterns of sentiment provided complementary information beyond cross-sectional sentiment measures. These analyses of longitudinal sentiment trajectories on Bluesky revealed that changes in sentiment over time were associated with current depressive symptom severity and age at diagnosis but not with diagnostic status itself; however, these results for depressive symptom severity did not remain statistically significant after FDR correction, whereas the associations with age at time of diagnosis remained significant. In particular, later age of depression diagnosis was associated with both lower baseline sentiment and steeper sentiment change over time, whereas binary diagnostic status showed little correspondence with sentiment dynamics. These exploratory findings suggest that longitudinal patterns of sentiment may capture depression heterogeneity that is not reflected by symptom measures or diagnostic labels alone [32,33]. Although linear trends explained limited variance in within-person sentiment, the pattern we observed is consistent with the episodic and context-dependent nature of sentiment in naturalistic social media data and underscores the importance of modeling trajectories rather than relying solely on aggregate sentiment measures [34].

Our findings also showed differences between platforms that should be considered for future research. Bluesky, which offers a microblogging format where individuals may post their beliefs and interact with other users, yielded slightly different patterns from those of Reddit, which offers a long-form, topic-centered discussion forum. Prior research has shown that the format of social media impacts how much time people spend on it, and individuals can become addicted to social networking platforms [35-38]. Prior work also shows a significant relationship between time spent on social media and declining mental health [39], although most of this work has been conducted in adolescent samples. Furthermore, research shows that negative thinking is contagious [40,41]. The dynamic relationships among distorted content, contagious negative thinking, and depression symptoms remain to be tested in midlife and older adults across different social media platforms.

On Bluesky, users who had ever had depression showed lower VADER compound sentiment and lower CDS prevalence than users who had never had depression in unadjusted analyses. However, the CDS association did not remain significant after adjustment for age and sex. On Reddit, depression diagnosis was not associated with CDS prevalence. The platform-specific CDS findings may reflect differences in platform structure, language use, or sample composition. First, most work on social media involves young adults and not adults aged 50 years and older, whose online language use and disclosure may be systematically different from those of young people. For example, in one study using MySpace comparing teenagers (aged 13-19 years) to older adults (aged ≥60 years), teenagers used more self-references and negative emotions [42]. However, results likely vary by platform. Next, there are sampling challenges in recruiting adults who use social media and are willing to share their data, which leads to self-selected groups of people with more symptoms or who more willingly self-disclose their social media timelines [43,44]. Self-disclosure on social media is widely studied among adolescents and young people, but less is known about self-disclosure among midlife and older adults [45,46]. While some studies have been conducted, little is known about how midlife and older adults express psychological distress online or how their online language can serve as an early warning sign of risk. Some recent work suggests that there is a positive association between internet use and mental health in midlife and older adults [47], but more work is needed to understand this.

This work has broader implications for cognitive aging research in the digital age. Epidemiological studies suggest an association between depression and Alzheimer disease and related dementias (ADRD) [48]. Even earlier-life depression increases risk of dementia later in life [49]. Unrecognized or untreated depression in older adults is an urgent public health concern and critical intervention target. An initial step in examining the relationship between depression and ADRD is to understand depression in midlife and older adults using digital tools to identify and quantify depression risk factors. Digital language markers may offer a scalable and low-burden tool to characterize depressive symptoms in midlife and older adults and potentially identify early vulnerability to cognitive decline. However, the clinical validity and predictive utility of these digital language markers require additional study. The current work provides a step toward integrating digital phenotyping with established epidemiological and clinical approaches to an advanced understanding of the relationship between depression and ADRD.

Despite its strengths, including a novel focus on sentiment and cognitive distortions in adults 50 years and older and a vetted, theory-driven approach for cognitive distortion detection, there are limitations to the current work. First, the samples were small in comparison to our prior work with Twitter and large-scale adolescent studies, so further work is needed to generalize our findings. Second, our sample was obtained via a self-report survey where individuals disclosed a diagnosis of depression and reported on their current symptoms of depression. Thus, there is a sampling bias [50] whereby we only recruited users who were willing to disclose their diagnostic status and share their social media timelines. Moreover, some participants provided handles for both platforms and, therefore, contributed language to both the Reddit and Bluesky datasets. Because analyses were conducted separately within each platform, this is unlikely to impact our results, but the overlap of platform samples may reduce the independence of our comparisons across platforms and should be considered when interpreting differences between Reddit and Bluesky. Additionally, because time-stamped post-level data were unavailable for Reddit at the time of analysis, longitudinal sentiment analysis could only be conducted for Bluesky, which limited our ability to compare temporal patterns in sentiment across platforms.

Future work should prioritize several directions. First, linking social media timelines to independently assessed clinical data would allow for stronger validation of linguistic markers of depression in older adults. Clinical data could include clinician-verified depression diagnoses, symptom history, treatment history, and cognitive performance. Linking social media timelines to clinical data could help determine whether language-based markers correspond to clinical indicators. Second, studying variation in the language used by individuals online could help understand cognitive decline. By integrating NLP and deep learning, this work could test whether language-based markers (cognitive distortions and perplexity) are enriched among older adults at elevated depression risk, positioning them as candidate indicators of cognitive vulnerability for future validation [51]. Still, there are many trade-offs between machine learning and deep learning for mental illness detection on social media, including accuracy, interpretability, and computational efficiency [52]. We believe that the current approach is innovative and scalable and leverages naturally occurring online data to identify early warning signs in at-risk populations.

In summary, the current study provides a proof of concept for using social media language from Bluesky and Reddit to detect language sentiment and cognitive distortions in midlife and older adults, a group that remains comparatively underrepresented in digital mental health research. This study lays the groundwork for future work that links online behavior to cognitive and clinical outcomes. Our findings support further study of how sentiment in online language relates to depression history and current depressive symptoms among adults aged 50 years and older. Larger longitudinal samples are needed before these features can be considered for detection or prediction.

## Supplementary material

10.2196/95023 Multimedia Appendix 1 Supplemental materials.

## Abbreviations

ADRD: Alzheimer disease and related dementias
CDS: cognitive distortion schemata
FDR: false discovery rate
NLP: natural language processing
PHQ-8: Patient Health Questionnaire–8
VADER: Valence Aware Dictionary and Sentiment Reasoner
