# Supplementary material for: Naturalistic Online Language as a Marker of Depression in Midlife and Older Adults: Computational Text Analysis of Bluesky and Reddit Posts
Source: J Med Internet Res. 2026 Aug 26;28:e95023. doi: 10.2196/95023 (PMC13510638; doi:10.2196/95023)
Supplement: Supplementary file 1 [file jmir-v28-e95023-s001.docx]

SI Table 1: Bootstrapped pairwise correlation statistics for Bluesky data

|  | Variable 1 | Variable 2 | ρ (bootstrap mean) | 95% CI | p | p (FDR) |
| --- | --- | --- | --- | --- | --- | --- |
| 0 | CDS Prevalence | PHQ-8 Total | 0.124 | [-0.030, 0.276] | 0.119 | 0.198 |
| 1 | CDS Prevalence | Diagnosed | -0.171 | [-0.234, -0.106] | <.001 | 0 |
| 2 | CDS Prevalence | Age | 0.018 | [-0.142, 0.175] | 0.82 | 0.838 |
| 3 | CDS Prevalence | Diagnosis Age | -0.094 | [-0.279, 0.095] | 0.32 | 0.411 |
| 4 | CDS Prevalence | vader_neg | 0.167 | [0.089, 0.240] | <.001 | 0 |
| 5 | CDS Prevalence | vader_neu | 0.063 | [-0.014, 0.142] | 0.084 | 0.169 |
| 6 | CDS Prevalence | vader_pos | -0.115 | [-0.190, -0.038] | 0.001 | 0.004 |
| 7 | CDS Prevalence | vader_comp | 0.09 | [0.014, 0.169] | 0.012 | 0.031 |
| 8 | CDS Prevalence | vader_comp_var | 0.526 | [0.458, 0.590] | <.001 | 0 |
| 9 | PHQ-8 Total | Diagnosed | 0.549 | [0.419, 0.660] | <.001 | 0 |
| 10 | PHQ-8 Total | Age | -0.176 | [-0.328, -0.014] | 0.026 | 0.058 |
| 11 | PHQ-8 Total | Diagnosis Age | 0.034 | [-0.156, 0.221] | 0.717 | 0.75 |
| 12 | PHQ-8 Total | vader_neg | 0.043 | [-0.117, 0.201] | 0.585 | 0.675 |
| 13 | PHQ-8 Total | vader_neu | -0.092 | [-0.252, 0.070] | 0.244 | 0.338 |
| 14 | PHQ-8 Total | vader_pos | 0.07 | [-0.087, 0.226] | 0.376 | 0.47 |
| 15 | PHQ-8 Total | vader_comp | 0.034 | [-0.126, 0.192] | 0.669 | 0.735 |
| 16 | PHQ-8 Total | vader_comp_var | 0.108 | [-0.060, 0.273] | 0.177 | 0.284 |
| 17 | Diagnosed | Diagnosis Age | 0 | [0.000, 0.000] | 1 | 1 |
| 18 | Diagnosed | vader_neg | 0.065 | [-0.013, 0.143] | 0.072 | 0.155 |
| 19 | Diagnosed | vader_neu | -0.062 | [-0.132, 0.010] | 0.089 | 0.169 |
| 20 | Diagnosed | vader_pos | -0.041 | [-0.112, 0.030] | 0.261 | 0.346 |
| 21 | Diagnosed | vader_comp | -0.165 | [-0.233, -0.095] | <.001 | <.001 |
| 22 | Diagnosed | vader_comp_var | -0.125 | [-0.195, -0.053] | 0.001 | 0.002 |
| 23 | Age | Diagnosed | -0.232 | [-0.372, -0.085] | 0.003 | 0.008 |
| 24 | Age | Diagnosis Age | 0.366 | [0.202, 0.513] | <.001 | <.001 |
| 25 | Age | vader_neg | -0.029 | [-0.178, 0.118] | 0.714 | 0.75 |
| 26 | Age | vader_neu | 0.104 | [-0.051, 0.257] | 0.189 | 0.293 |
| 27 | Age | vader_pos | -0.101 | [-0.251, 0.056] | 0.204 | 0.306 |
| 28 | Age | vader_comp | -0.092 | [-0.244, 0.063] | 0.248 | 0.338 |
| 29 | Age | vader_comp_var | -0.178 | [-0.330, -0.016] | 0.025 | 0.058 |
| 30 | Diagnosis Age | vader_neg | -0.147 | [-0.326, 0.042] | 0.118 | 0.198 |
| 31 | Diagnosis Age | vader_neu | 0.158 | [-0.019, 0.331] | 0.091 | 0.169 |
| 32 | Diagnosis Age | vader_pos | -0.079 | [-0.253, 0.097] | 0.399 | 0.485 |
| 33 | Diagnosis Age | vader_comp | 0.041 | [-0.147, 0.232] | 0.666 | 0.735 |
| 34 | Diagnosis Age | vader_comp_var | -0.111 | [-0.285, 0.065] | 0.24 | 0.338 |
| 35 | vader_neg | vader_neu | -0.238 | [-0.314, -0.162] | <.001 | <.001 |
| 36 | vader_neg | vader_pos | -0.23 | [-0.304, -0.154] | <.001 | <.001 |
| 37 | vader_neu | vader_pos | -0.8 | [-0.839, -0.755] | <.001 | <.001 |
| 38 | vader_comp | vader_neg | -0.606 | [-0.658, -0.549] | <.001 | <.001 |
| 39 | vader_comp | vader_neu | -0.303 | [-0.373, -0.228] | <.001 | <.001 |
| 40 | vader_comp | vader_pos | 0.679 | [0.632, 0.722] | <.001 | <.001 |
| 41 | vader_comp | vader_comp_var | -0.021 | [-0.098, 0.058] | 0.569 | 0.674 |
| 42 | vader_comp_var | vader_neg | 0.457 | [0.387, 0.523] | <.001 | <.001 |
| 43 | vader_comp_var | vader_neu | -0.182 | [-0.258, -0.104] | <.001 | <.001 |
| 44 | vader_comp_var | vader_pos | 0.061 | [-0.019, 0.140] | 0.094 | 0.169 |

SI Table 2: Bootstrapped pairwise correlation statistics for Reddit data

|  | Variable 1 | Variable 2 | ρ (bootstrap mean) | 95% CI | p | p (FDR) |
| --- | --- | --- | --- | --- | --- | --- |
| 0 | CDS Prevalence | PHQ-8 Total | 0.037 | [-0.042, 0.113] | 0.373 | 0.509 |
| 1 | CDS Prevalence | Diagnosed | 0.019 | [-0.062, 0.098] | 0.641 | 0.74 |
| 2 | CDS Prevalence | Age | -0.03 | [-0.107, 0.048] | 0.471 | 0.624 |
| 3 | CDS Prevalence | Diagnosis Age | 0.052 | [-0.038, 0.145] | 0.277 | 0.416 |
| 4 | CDS Prevalence | vader_neg | 0.159 | [0.075, 0.244] | <.001 | <.001 |
| 5 | CDS Prevalence | vader_neu | 0.121 | [0.034, 0.208] | 0.003 | 0.007 |
| 6 | CDS Prevalence | vader_pos | -0.19 | [-0.271, -0.107] | <.001 | <.001 |
| 7 | CDS Prevalence | vader_comp | 0.072 | [-0.016, 0.158] | 0.079 | 0.155 |
| 8 | CDS Prevalence | vader_comp_var | 0.538 | [0.463, 0.608] | <.001 | <.001 |
| 9 | PHQ-8 Total | Diagnosed | 0.534 | [0.474, 0.589] | <.001 | <.001 |
| 10 | PHQ-8 Total | Age | -0.141 | [-0.220, -0.061] | 0.001 | 0.002 |
| 11 | PHQ-8 Total | Diagnosis Age | -0.052 | [-0.147, 0.042] | 0.277 | 0.416 |
| 12 | PHQ-8 Total | vader_neg | 0.11 | [0.030, 0.188] | 0.007 | 0.018 |
| 13 | PHQ-8 Total | vader_neu | -0.081 | [-0.160, -0.001] | 0.05 | 0.101 |
| 14 | PHQ-8 Total | vader_pos | -0.01 | [-0.090, 0.071] | 0.815 | 0.853 |
| 15 | PHQ-8 Total | vader_comp | -0.088 | [-0.167, -0.008] | 0.032 | 0.069 |
| 16 | PHQ-8 Total | vader_comp_var | 0.125 | [0.044, 0.203] | 0.003 | 0.007 |
| 17 | Diagnosed | Diagnosis Age | 0 | [0.000, 0.000] | 1 | 1 |
| 18 | Diagnosed | vader_neg | 0.025 | [-0.055, 0.101] | 0.533 | 0.642 |
| 19 | Diagnosed | vader_neu | -0.058 | [-0.136, 0.022] | 0.156 | 0.271 |
| 20 | Diagnosed | vader_pos | 0.017 | [-0.061, 0.098] | 0.671 | 0.754 |
| 21 | Diagnosed | vader_comp | -0.049 | [-0.125, 0.030] | 0.233 | 0.375 |
| 22 | Diagnosed | vader_comp_var | 0.069 | [-0.010, 0.147] | 0.096 | 0.173 |
| 23 | Age | Diagnosed | -0.193 | [-0.273, -0.111] | <.001 | <.001 |
| 24 | Age | Diagnosis Age | 0.235 | [0.143, 0.324] | <.001 | <.001 |
| 25 | Age | vader_neg | 0.025 | [-0.055, 0.102] | 0.542 | 0.642 |
| 26 | Age | vader_neu | -0.015 | [-0.098, 0.063] | 0.708 | 0.777 |
| 27 | Age | vader_pos | 0.005 | [-0.075, 0.086] | 0.908 | 0.928 |
| 28 | Age | vader_comp | -0.026 | [-0.104, 0.056] | 0.534 | 0.642 |
| 29 | Age | vader_comp_var | -0.028 | [-0.110, 0.054] | 0.495 | 0.637 |
| 30 | Diagnosis Age | vader_neg | -0.083 | [-0.176, 0.015] | 0.085 | 0.159 |
| 31 | Diagnosis Age | vader_neu | 0.158 | [0.069, 0.243] | 0.001 | 0.003 |
| 32 | Diagnosis Age | vader_pos | -0.122 | [-0.213, -0.029] | 0.011 | 0.024 |
| 33 | Diagnosis Age | vader_comp | -0.05 | [-0.146, 0.049] | 0.297 | 0.431 |
| 34 | Diagnosis Age | vader_comp_var | -0.048 | [-0.143, 0.050] | 0.322 | 0.452 |
| 35 | vader_neg | vader_neu | -0.238 | [-0.321, -0.151] | <.001 | <.001 |
| 36 | vader_neg | vader_pos | -0.215 | [-0.298, -0.130] | <.001 | <.001 |
| 37 | vader_neu | vader_pos | -0.829 | [-0.869, -0.782] | <.001 | <.001 |
| 38 | vader_comp | vader_neg | -0.583 | [-0.641, -0.519] | <.001 | <.001 |
| 39 | vader_comp | vader_neu | -0.336 | [-0.416, -0.255] | <.001 | <.001 |
| 40 | vader_comp | vader_pos | 0.663 | [0.607, 0.714] | <.001 | <.001 |
| 41 | vader_comp | vader_comp_var | -0.051 | [-0.141, 0.037] | 0.218 | 0.364 |
| 42 | vader_comp_var | vader_neg | 0.482 | [0.407, 0.554] | <.001 | <.001 |
| 43 | vader_comp_var | vader_neu | -0.177 | [-0.260, -0.091] | <.001 | <.001 |
| 44 | vader_comp_var | vader_pos | 0.013 | [-0.075, 0.101] | 0.746 | 0.799 |

SI Table 3: Bootstrapped correlation statistics for linear model fit

|  | Variable 1 | Variable 2 | ρ (bootstrap mean) | 95% CI | p | p (FDR) |
| --- | --- | --- | --- | --- | --- | --- |
| 0 | R² | PHQ-8 Total | 0.025 | [-0.126, 0.175] | 0.754 | 0.823 |
| 1 | R² | Age | 0.05 | [-0.105, 0.202] | 0.531 | 0.823 |
| 2 | R² | Diagnosed | 0.006 | [-0.152, 0.162] | 0.943 | 0.943 |
| 3 | R² | Diagnosis Age | -0.04 | [-0.227, 0.153] | 0.674 | 0.823 |
| 4 | Intercept | PHQ-8 Total | -0.122 | [-0.278, 0.038] | 0.126 | 0.378 |
| 5 | Intercept | Age | -0.045 | [-0.203, 0.112] | 0.576 | 0.823 |
| 6 | Intercept | Diagnosed | -0.025 | [-0.184, 0.133] | 0.754 | 0.823 |
| 7 | Intercept | Diagnosis Age | -0.271 | [-0.434, -0.101] | 0.004 | 0.027 |
| 8 | Slope | PHQ-8 Total | 0.189 | [0.030, 0.338] | 0.018 | 0.07 |
| 9 | Slope | Age | 0.049 | [-0.107, 0.204] | 0.545 | 0.823 |
| 10 | Slope | Diagnosed | 0.059 | [-0.093, 0.216] | 0.461 | 0.823 |
| 11 | Slope | Diagnosis Age | 0.265 | [0.094, 0.424] | 0.004 | 0.027 |

SI Table 4: PHQ8 scores by platform and diagnosis status

| Platform, Diagnosis Status | count | PHQ8 mean | SD | min | 25% | 50% | 75% | max |
| --- | --- | --- | --- | --- | --- | --- | --- | --- |
| Bluesky, never-diagnosed | 56 | 3.46 | 4.19 | 0 | 0 | 2 | 5 | 18 |
| Bluesky, ever-diagnosed | 154 | 10.01 | 5.27 | 0 | 6 | 9.5 | 14 | 24 |
| Reddit, never-diagnosed | 182 | 3.40 | 3.89 | 0 | 1 | 2 | 4 | 20 |
| Reddit, ever-diagnosed | 506 | 10.26 | 5.76 | 0 | 6 | 10 | 14 | 24 |

SI Table 5: Bluesky sensitivity analysis. Correlation statistics computed for populations with increasing thresholds for number of posts.

| Min. Posts | N Users | Predictor | Outcome | ρ | p | p (FDR) |
| --- | --- | --- | --- | --- | --- | --- |
| 1 | 210 | PHQ8_total | CDS_prevalence | 0.124 | 0.119 | 0.158 |
| 1 | 210 | PHQ8_total | vader_comp | 0.034 | 0.669 | 0.669 |
| 1 | 210 | diagnosed | CDS_prevalence | -0.171 | <.001 | <.001 |
| 1 | 210 | diagnosed | vader_comp | -0.165 | <.001 | <.001 |
| 5 | 161 | PHQ8_total | CDS_prevalence | 0.122 | 0.146 | 0.194 |
| 5 | 161 | PHQ8_total | vader_comp | 0.041 | 0.629 | 0.629 |
| 5 | 161 | diagnosed | CDS_prevalence | -0.188 | <.001 | <.001 |
| 5 | 161 | diagnosed | vader_comp | -0.184 | <.001 | <.001 |
| 10 | 135 | PHQ8_total | CDS_prevalence | 0.125 | 0.158 | 0.211 |
| 10 | 135 | PHQ8_total | vader_comp | 0.033 | 0.712 | 0.712 |
| 10 | 135 | diagnosed | CDS_prevalence | -0.198 | <.001 | <.001 |
| 10 | 135 | diagnosed | vader_comp | -0.203 | <.001 | <.001 |
| 25 | 103 | PHQ8_total | CDS_prevalence | 0.076 | 0.445 | 0.594 |
| 25 | 103 | PHQ8_total | vader_comp | -0.027 | 0.786 | 0.786 |
| 25 | 103 | diagnosed | CDS_prevalence | -0.218 | <.001 | <.001 |
| 25 | 103 | diagnosed | vader_comp | -0.238 | <.001 | <.001 |
| 50 | 80 | PHQ8_total | CDS_prevalence | 0.244 | 0.03 | 0.04 |
| 50 | 80 | PHQ8_total | vader_comp | 0.086 | 0.451 | 0.451 |
| 50 | 80 | diagnosed | CDS_prevalence | -0.202 | <.001 | <.001 |
| 50 | 80 | diagnosed | vader_comp | -0.246 | <.001 | <.001 |

SI Table 6: Reddit sensitivity analysis. Correlation statistics computed for populations with increasing thresholds for number of posts.

| Min. Posts | N Users | Predictor | Outcome | ρ | p | p (FDR) |
| --- | --- | --- | --- | --- | --- | --- |
| 1 | 686 | PHQ8_total | CDS_prevalence | 0.036 | 0.378 | 0.504 |
| 1 | 686 | PHQ8_total | vader_comp | -0.086 | 0.036 | 0.143 |
| 1 | 686 | diagnosed | CDS_prevalence | 0.024 | 0.557 | 0.557 |
| 1 | 686 | diagnosed | vader_comp | -0.052 | 0.201 | 0.403 |
| 5 | 565 | PHQ8_total | CDS_prevalence | 0.034 | 0.424 | 0.565 |
| 5 | 565 | PHQ8_total | vader_comp | -0.079 | 0.067 | 0.268 |
| 5 | 565 | diagnosed | CDS_prevalence | 0.001 | 0.988 | 0.988 |
| 5 | 565 | diagnosed | vader_comp | -0.05 | 0.239 | 0.478 |
| 10 | 505 | PHQ8_total | CDS_prevalence | 0.037 | 0.417 | 0.556 |
| 10 | 505 | PHQ8_total | vader_comp | -0.084 | 0.06 | 0.24 |
| 10 | 505 | diagnosed | CDS_prevalence | -0.013 | 0.768 | 0.768 |
| 10 | 505 | diagnosed | vader_comp | -0.059 | 0.188 | 0.376 |
| 25 | 409 | PHQ8_total | CDS_prevalence | 0.022 | 0.653 | 0.653 |
| 25 | 409 | PHQ8_total | vader_comp | -0.07 | 0.158 | 0.51 |
| 25 | 409 | diagnosed | CDS_prevalence | -0.056 | 0.255 | 0.51 |
| 25 | 409 | diagnosed | vader_comp | -0.034 | 0.491 | 0.653 |
| 50 | 342 | PHQ8_total | CDS_prevalence | 0.048 | 0.38 | 0.59 |
| 50 | 342 | PHQ8_total | vader_comp | -0.042 | 0.442 | 0.59 |
| 50 | 342 | diagnosed | CDS_prevalence | -0.051 | 0.347 | 0.59 |
| 50 | 342 | diagnosed | vader_comp | -0.007 | 0.902 | 0.902 |

SI Table 7: Partial Spearman correlations between depression indicators (PHQ-8 total score, diagnosis status) and linguistic markers (CDS prevalence, VADER compound sentiment) controlling for age and biological sex with FDR correction for 8 tests.

| Platform | Predictor | Outcome | Partial ρ | 95% CI | p | p (FDR) |
| --- | --- | --- | --- | --- | --- | --- |
| bsky | PHQ8_total | CDS_prevalence | 0.044 | [-0.090, 0.180] | 0.531 | 0.856 |
| bsky | diagnosed | CDS_prevalence | -0.038 | [-0.170, 0.100] | 0.585 | 0.856 |
| bsky | PHQ8_total | vader_comp | 0.024 | [-0.110, 0.160] | 0.734 | 0.856 |
| bsky | diagnosed | vader_comp | 0.022 | [-0.110, 0.160] | 0.749 | 0.856 |
| reddit | PHQ8_total | CDS_prevalence | 0.047 | [-0.030, 0.120] | 0.218 | 0.581 |
| reddit | diagnosed | CDS_prevalence | -0.003 | [-0.080, 0.070] | 0.942 | 0.942 |
| reddit | PHQ8_total | vader_comp | -0.066 | [-0.140, 0.010] | 0.085 | 0.34 |
| reddit | diagnosed | vader_comp | -0.07 | [-0.140, 0.010] | 0.068 | 0.34 |

SI Table 8: Depression status and severity and VADER compound sentiment summary.

|  | Diagnosed vs. Vader Compound | PHQ-8 vs. Vader Compound |
| --- | --- | --- |
| Bluesky | ρ = -0.16 (P<.001) | ρ = 0.03 (P=.67) |
| Reddit | ρ = -0.05 (P=.23) | ρ = -0.09 (P=.03) |
